# Timing of Tributyrin Supplementation Differentially Modulates Gastrointestinal Inflammation and Gut Microbial Recolonization Following Murine Ileocecal Resection

**DOI:** 10.3390/nu13062069

**Published:** 2021-06-17

**Authors:** Valentin Mocanu, Heekuk Park, Jerry Dang, Naomi Hotte, Aducio Thiesen, Michael Laffin, Haili Wang, Daniel Birch, Karen Madsen

**Affiliations:** 1 Department of Surgery, University of Alberta, Edmonton, AB T6G 2R3, Canada; dang2@ualberta.ca (J.D.); mlaffin@ualberta.ca (M.L.); haili@ualberta.ca (H.W.); dbirch@ualberta.ca (D.B.); 2 Columbia University Medical Center, New York, NY 10032, USA; hp2523@cumc.columbia.edu; 3 Department of Medicine, University of Alberta, Edmonton, AB T6G 2R3, Canada; nhotte@ualberta.ca; 4 Department of Laboratory Medicine and Pathology, University of Alberta, Edmonton, AB T6G 2R3, Canada; athiesen@ualberta.ca; 5 Division of Gastroenterology, University of Alberta, Edmonton, AB T6G 2R3, Canada; kmadsen@ualberta.ca

**Keywords:** tributyrin, inflammatory bowel disease, ileocecal resection, Crohn’s disease, microbiome

## Abstract

Background: Gastrointestinal surgery imparts dramatic and lasting imbalances, or dysbiosis, to the composition of finely tuned microbial ecosystems. The aim of the present study was to use a mouse ileocecal resection (ICR) model to determine if tributyrin (TBT) supplementation could prevent the onset of microbial dysbiosis or alternatively enhance the recovery of the gut microbiota and reduce gastrointestinal inflammation. Methods: Male wild-type (129 s1/SvlmJ) mice aged 8–15 weeks were separated into single cages and randomized 1:1:1:1 to each of the four experimental groups: control (CTR), preoperative TBT supplementation (PRE), postoperative TBT supplementation (POS), and combined pre- and postoperative supplementation (TOT). ICR was performed one week from baseline assessment with mice assessed at 1, 2, 3, and 4 weeks postoperatively. Primary outcomes included evaluating changes to gut microbial communities occurring from ICR to 4 weeks. Results: A total of 34 mice that underwent ICR (CTR *n* = 9; PRE *n* = 10; POS *n* = 9; TOT *n* = 6) and reached the primary endpoint were included in the analysis. Postoperative TBT supplementation was associated with an increased recolonization and abundance of anaerobic taxa including *Bacteroides thetaiotomicorn, Bacteroides caecimuris, Parabacteroides distasonis,* and *Clostridia*. The microbial recolonization of PRE mice was characterized by a bloom of aerotolerant organisms including *Staphylococcus, Lactobacillus, Enteroccaceae,* and *Peptostreptococcacea.* PRE mice had a trend towards decreased ileal inflammation as evidenced by decreased levels of IL-1β (*p* = 0.09), IL-6 (*p* = 0.03), and TNF-α (*p* < 0.05) compared with mice receiving TBT postoperatively. In contrast, POS mice had trends towards reduced colonic inflammation demonstrated by decreased levels of IL-6 (*p* = 0.07) and TNF-α (*p* = 0.07). These changes occurred in the absence of changes to fecal short-chain fatty acid concentrations or histologic injury scoring. Conclusions: Taken together, the results of our work demonstrate that the timing of tributyrin supplementation differentially modulates gastrointestinal inflammation and gut microbial recolonization following murine ICR.

## 1. Introduction

The human gut microbiome contains over 100 trillion microorganisms, species which are not simply innocent bystanders but have co-evolved with their human hosts to achieve a complex symbiotic relationship integral to human health [1,2,3,4,5,6]. In a healthy state, commensal bacteria provide a number of essential functions from producing anti-inflammatory short-chain fatty acid (SCFA) metabolites to regulating both adaptive and innate immunity, conjugating bile acids, maintaining gut barrier integrity, synthesizing antimicrobial peptides, and providing colonization resistance to gut pathogens [2,7,8].

Gastrointestinal surgery imparts dramatic and lasting imbalances, or dysbiosis, to the composition of these finely tuned microbial ecosystems [9,10,11]. Luminal exposure to oxygen facilitates a depletion of anti-inflammatory obligate anaerobes and a bloom of pro-inflammatory aerotolerant organisms [12]. Animal models have demonstrated that perturbation of these commensal microbial communities triggers a loss of anti-inflammatory SCFAs, increased expression of bacterial virulence genes, and heightened protease activity [9,12,13]. The cumulative effects of these changes are important since they have been implicated in a variety of adverse clinical outcomes including surgical site infections, anastomotic leak, and postoperative recurrence of Crohn’s disease (CD).

Although our group and others have shown the importance of these microbial factors in mitigating adverse surgical outcomes and maintenance of host immune homeostasis in colitis models [10,11], it is not known whether this loss of microbial ecology can be avoided or the degree to which these shifts can be manipulated following surgery. Approaches that harness the gut microbiome in the immediate perioperative period thus present a novel opportunity to optimize the physiological stress imparted by surgery [14]. Current strategies including prebiotic, probiotic, and synbiotic supplementation, however, have been met with mixed success [10,15]. This is thought to be due to the inherent challenges of re-establishing anaerobic bacterial communities in an aerobic environment that is unable to successfully foster recolonization of native microbes [10,11,16].

An approach which instead emphasizes the re-establishment of an anaerobic and anti-inflammatory environment may thus prove more effective in restoring pre-surgical microbial ecology. A promising supplement to facilitate this recovery is tributyrin (TBT), a butyrate analogue which has drawn growing interest as a food additive [17] due to its ability to improve gut health by increasing butyrate delivery at the level of the colon and terminal [18]. Butyrate has been recently demonstrated to augment luminal hypoxia through modulation of hypoxia-inducible factor-1 (HIF-1) [19,20] and also to have anti-inflammatory effects [18,21]. In dextran sulphate sodium-induced colitis mice, HIF-1 has been found to play a protective role in the maintenance of gut barrier integrity through butyrate-mediated tight junction protein upregulation [22]. However, while TBT supplementation has recently demonstrated benefit in poultry [17] and piglet [23] gastrointestinal health, its effects on murine gut inflammation or microbial recolonization following surgery are currently not known.

To evaluate the above concept, the aim of the present study was to use an established mouse ileocecal resection (ICR) model to determine if perioperative TBT supplementation could prevent the onset of postoperative microbial dysbiosis or alternatively enhance the recovery of the gut microbiota and reduce gastrointestinal inflammation. ICR is the most commonly performed surgery in patients with CD and our murine ICR technique mirrors that performed in human patients. Therefore, the design of our study presents an excellent framework from which to translate basic biological findings to real-world clinical practice.

## 2. Materials and Methods

### 2.1. Experimental Design

Experimental protocols were approved by the University of Alberta animal ethics committee (AUP00000293) with perioperative mouse husbandry protocols approved by the university’s Health Sciences Laboratory Animal Services.

To evaluate for the inflammatory and microbial effects of perioperative tributyrin supplementation on a mouse model of ileocecal resection (ICR), a parallel four-arm study design was utilized. We chose ICR as our surgical model since our mouse ICR closely resembles the ICR performed in human patients. Since ICR is the most common surgery in patients with CD [11], this mouse model allows for feasible knowledge translation.

**Figure 1:**
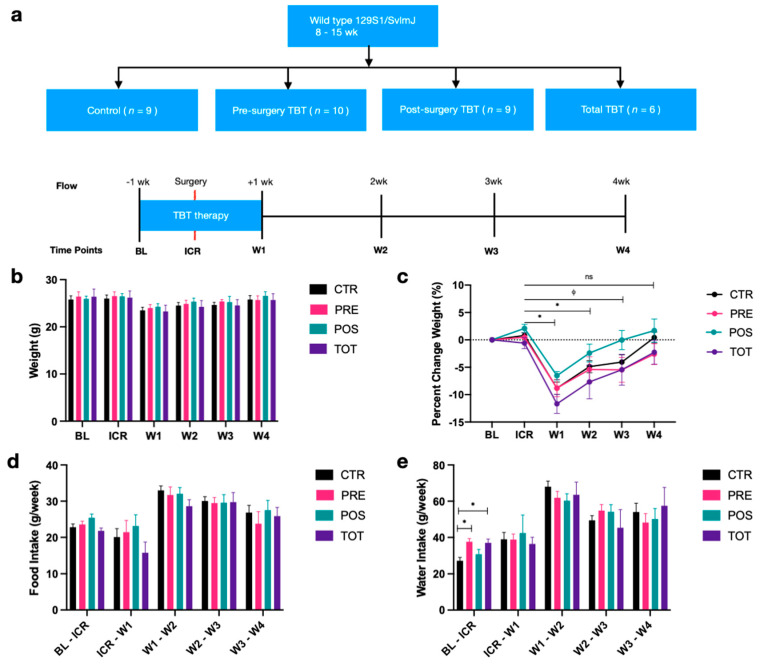
Overview of study design and changes in weight, food, and water intake. (**a**) Study design overview. Column graphs represent median +/− SEM. (**b**) Mouse weights from baseline to week 4 across intervention groups. (**c**) Percent change in weight relative to baseline across intervention groups. (**d**) Differences in weekly food intake across intervention groups. (**e**) Differences in weekly water intake across intervention groups. All *p*-values were two-sided with statistical significance defined as *p* < 0.05. * represents significance in paired analysis for percent change relative to baseline for all groups; ∅ represents significance in paired analysis for percent change relative to baseline for control group alone. ns represents values that are not significant.

The four intervention groups were control (CTR), preoperative TBT supplementation (PRE), postoperative TBT supplementation (POS), and combined pre- and postoperative supplementation (TOT) (Figure 1a). PRE mice received one week of preoperative TBT followed by ICR, POS mice received ICR followed by one week of postoperative TBT, and TOT mice received one week of preoperative TBT followed by ICR and an additional one week of postoperative TBT. CTR mice received surgery but no TBT supplementation.

At baseline (BL), male wild-type (129 s1/SvlmJ) mice aged 8–15 weeks were separated into single cages to avoid cage effects biasing microbial analysis and were randomized 1:1:1:1 to each of the four experimental groups. ICR was performed one week from baseline assessment, with mice assessed at 1, 2, 3, and 4 weeks postoperatively. Body weight along with water and chow consumption were measured at BL and then weekly until sacrifice. Stool samples were immediately collected and frozen at −80 °C at baseline, prior to start of the surgical liquid diet, and then weekly until our endpoint. At the start of four weeks, mice were sacrificed with the following collected and frozen at −80 °C for immunologic analysis: ocular blood, ileal tissue, colonic tissue, and anastomotic tissue.

### 2.2. Study Interventions

#### 2.2.1. Tributyrin Supplementation

All TBT intervention groups were provided tributyrin (Tributyrin 97% FG, Sigma Aldrich, Oakville, ON, Canada, Product# W222305) at a concentration of 10 mM. Rationale for TBT dosing was based on prior ethanol-induced gut injury mouse models which demonstrated evidence for improved gut epithelial integrity at the level of the ileum and proximal colon [18]. To minimize animal stress during the perioperative period, TBT supplementation was added to existing water bottles and provided ad libitum, while CTR mice received water alone in identical delivery systems. Standard mouse chow Labdiet 5001 (LabDiet, St. Louis, MO, USA) was further provided ad libitum throughout the course of the experiment, with the exception of the four perioperative days, where mice were kept on a liquid diet. Throughout, animals were housed in filter-top cages in humidity- and temperature-controlled facilities with regulated day/night cycles at the University of Alberta (Edmonton, AB, Canada).

#### 2.2.2. Ileocecal Resection

The ileocecal resection procedure was conducted using a modified version of a protocol previously described by our group [24]. Two days prior to ICR, mice were transitioned to a Lieber-DeCarli ‘82 liquid diet (Bio-Serv, Flemington, NJ, USA, Product#F1259SP) to minimize risk of postoperative obstruction. Following induction of anesthesia, meloxicam (Metacam^®^, 3 mg/kg) was administered subcutaneously. Induction and maintenance of anesthesia was achieved using isoflurane and titrated to a respiratory rate of approximately 50 breaths/min and absent pedal pain responses. A bolus of 30 mL/kg of normal saline was given subcutaneously, with the mice then positioned in a supine position and immobilized on a warming pad. Hair was clipped over the site of the incision and the abdomen was then prepped with betadine and draped with sterile gauze.

A 1 cm midline incision was made sharply and extended through the skin and peritoneum. The cecum was identified and delivered through the incision with the proximal colon and distal ileum. The ileocolic vascular pedicle was first identified and ligated. Next, the segmental blood supply to the terminal ileum located approximately 2 cm proximal to the ileocecal valve was ligated. The ischemic tissue was allowed to demarcate, and the colon and ileum were then transected at the borders of ischemia. The resulting ends of the colon and terminal ileum were inspected for perfusion, gently dilated, and then generally re-approximated on moistened sterile gauze using stay-sutures.

Using a dissecting microscope, an end-to-end anastomosis using simple interrupted 8-0 monofilament Prolene sutures was then created. Upon completion, the anastomosis was checked for leak and reinserted into the abdomen. The abdomen was irrigated with sterile saline (~2 mL) to ensure clear effluent. The abdominal wall and skin were closed in separate layers using 5-0 Vicryl in a running continuous fashion for the abdominal wall and in a simple interrupted fashion for the skin. Vet-bond (3M, Saint Paul, MN, USA) was applied to all external visible suture knots to prevent wound dehiscence.

Postoperatively, each mouse was recovered under a heat lamp until alert, placed in a clean cage, and maintained on a liquid diet for two days. Analgesia was continued for three days via daily meloxicam subcutaneous injections at 3 mg/kg. Water (CTR) and tributyrin supplementation were continued in the perioperative phase ad libitum. Solid chow diet was reintroduced two days postoperatively, during which animals were monitored twice daily for lack of stooling or other signs of obstruction. When followed closely, survival rates of this protocol approached 90%.

### 2.3. Primary and Secondary Outcomes

Primary outcomes included evaluating changes to gut microbial communities due to study intervention occurring from ICR to 4 weeks evaluated by 16S rRNA sequencing. Secondary outcomes included evaluating for differences in the following from ICR to 4 weeks: weight, water and food intake, inflammatory cytokines (LPS, IL-1β, IL-6, IL-10, TNF-α), fecal short-chain fatty acid concentrations, and histologic injury scoring.

### 2.4. Analytical Techniques

#### 2.4.1. Microbiome Extraction

Fecal DNA extraction for microbiome analysis was conducted using a modified MultiTarget Pharmaceuticals protocol. Bleached beads were added to tubes in combination with 200 μL of AquaStool (MultiTarget Pharmaceuticals, Colorado Springs, CO, USA), approximately 100 mg of thawed stool, and homogenized. The resultant homogenate was centrifuged (14,000× *g* for 5 min) followed by addition of 100 μL of AquaRemove (MultiTarget Pharmaceuticals, Colorado Springs, CO, USA). After recentrifugation, the supernatant was collected, and isopropanol was added prior to precipitation on ice for 10 min. The DNA pellet was collected and washed three times with 70% ethanol. A total of 100 μL of EB Buffer (Qiagen, Germantown, MD, USA) was then added to solubilize the DNA, followed by 1 μL of RNASE A (Qiagen, catalog 1007885). The mixture was incubated at 37 °C for 1 h and then recentrifuged. DNA precipitation was obtained with 10 μL of 5M NaCl, 100 μL of ice cold 100% ethanol, and a 30 min incubation at −20 °C. The mixture was recentrifuged and the pellet was rinsed three times with 70% ethanol. An additional 50 μL of EB buffer was added after removal of excess ethanol, and the solution was left overnight at 4 °C for solubilization.

After ensuring appropriate extraction quality using a Nanodrop 1000 Series device (Thermo Fisher Scientific, Waltham, MA, USA), samples were sent for 16S rRNA gene amplicon sequencing (Genome Canada, Montreal, QC, Canada). Microbial composition was characterized by 16S rRNA gene amplicon sequencing of the v4 region using MiSeq Illumina technology (2 × 300 bp) and the following forward and reverse primers: 341F ‘CCTACGGGNGGCWGCAGTCCTACGGGNGGCWGCAGACCCTACGGGNGGCWGCAGCTACCTACGGGNGGCWGCAG’ and 805R ‘GACTACHVGGGTATCTAATCCTGACTACHVGGGTATCTAATCCACGACTACHVGGGTATCTAATCCCTAGACTACHVGGGTATCTAATCC’.

#### 2.4.2. Enzyme-Linked Immunosorbent Assay (ELISA)

Assays were conducted in singlet using commercially available kits while following manufacturer protocol and storage recommendations. Preparation of frozen tissue homogenate for ELISA analysis was performed by combining the frozen tissue together with 0.5 mm silica beads (BioSpec Products, Bartlesville, OK, USA, catalog 11079105) and 400 μL of an extraction buffer mixture of Tween-20 (0.05%), Bovine Serum Albumin (BSA) 0.1%, and 1 μL/mL protease inhibitor (Sigma P-8340) solubilized in 1x phosphate-buffered saline (PBS). Samples were then homogenized with a beadmill (MPBiologicals, Fast-Prep 24) for 40 s at 6 m/s and centrifuged at 10,000× *g* to pellet excess debris. Resulting supernatant was then used for ELISA analysis with concentrations corrected for dry weight of tissue. Immunologic evaluation included IL-1β (R&D Systems DuoSet ELISA, catalog DY201-05), IL-6 (R&D Systems DuoSet ELISA, catalog DY206-05), TNF-α (R&D Systems DuoSet ELISA, catalog DY210-05), and LPS (Abbexa Endotoxin (ET) ELISA Kit, catalog ABX514093).

#### 2.4.3. SCFA Extraction

Fecal short-chain fatty acid concentrations were analyzed using gas chromatography at the Agricultural, Food and Nutritional Science chromatography core facility as previously described [10]. Briefly, 800 μL of 0.1 N hydrochloric acid and 200 μL of 25% phosphoric acid were added to approximately 0.2 g of stool. The contents were vortexed until fully homogenized and centrifuged at 5000× *g* for 15 min or until a clear supernatant was obtained. An internal standard solution (150 mg of 4-methyl-valeric acid, S381810, Sigma-Aldrich), 5% phosphoric acid, and supernatant was then added to glass chromatography tubes and stored at −80 °C prior to analysis. Samples were analyzed with a gas chromatograph (Bruker SCION 456-GC, Bruker Corporation, Billerica, MA, USA) using a 30 m × 0.53 mm inner diameter × 0.5 μm film thickness capillary column (Stabilwax-DA, Restek Corporation, Belefonte, PA, USA).

#### 2.4.4. Histology Preparation and Analysis

At the time of sacrifice, sections of perianastomotic ileum, colon, and anastomotic tissue were opened and flushed with 1xPBS+Gentamycin 50 μg/mL (Gibco 15750-60), then fixed in 10% buffered formalin (Fisher Scientific #245-685) for a minimum of 24 h. Tissues were processed for paraffin embedding using a Leica Automated Tissue Processor with the following program: 1 h 70% ethanol, 1 h 90% ethanol, 3 × 30 min 100% ethanol, 4 × 40 min Xylene, then 2 × 40 h paraffin under vacuum. Embedded tissues were further cut and processed for Hematoxylin & Eosin staining at the Alberta Diabetes Institute histology with their standard procedures. Slide were blinded and scored by a single pathologist (AT) using a validated scale which evaluates enterocyte injury, epithelial hyperplasia, lamina propria, lymphocytes, and lamina propria neutrophils [25].

### 2.5. Statistical Analysis

Continuous variables were reported as means ± standard deviations if normally distributed or medians and interquartile ranges if non-normally distributed. Categorical data were reported as proportions and analyzed using the Cochran–Mantel–Haenszel^2^ test. Within-group paired changes were assessed using two-tailed Wilcoxon signed-rank test while between-group comparisons were assessed using the Mann–Whitney *U* test. Outliers were defined as greater than three standard deviations and were removed prior to analysis. Analyses for changes in weight, dietary intake, and immunologic data were conducted using STATA 15 (StataCorp 2017; College Station, TX, USA). Figures were designed using Prism 9.0.0 (GraphPad Software, San Diego, CA, USA). Statistical significance was defined using two-tailed tests with a *p* value < 0.05.

For microbial analysis, 16S rRNA sequences were first processed using the divisive amplicon de-noising algorithm version 2 (DADA2) pipeline. This pipeline allowed for sequencing quality control and was used for trimming, error correction, exact sequence inference, chimera removal, and for generation of an amplicon sequence variant (ASV) table. Taxonomic classification was performed using a native RDP Bayesian classifier alongside the Silva database (version 138). Calculation of α-diversity (Shannon, Chao) and β-diversity (weighted UniFrac) was performed using the ‘phyloseq’ (v1.28.0) package in R. Samples with a minimum cut-off of 10,000 counts based on α-diversity rarefaction or where rarefaction curves plateaued were included for analysis. Changes in β-diversity were evaluated using the permutational multivariate analysis of variance (PERMANOVA), a non-parametric test which determines if the centroids of sample clusters differ. Differences in bacterial ASV abundance were analyzed using DESeq2, an estimate of variance–mean dependence based on a negative binomial distribution model. Microbial analysis was conducted using R (Version 3.5.1).

## 3. Results

### 3.1. Tributyrin Is Associated with a Quicker Restoration of Postoperative Weight Loss

A total of 34 mice that underwent ICR (CTR *n* = 9; PRE *n* = 10; POS *n* = 9; TOT *n* = 6) and reached the primary endpoint were included in the analysis. There were no differences for mouse weight (Figure 1b) or food intake (Figure 1d) between groups either at baseline or across any other time points. PRE and TOT mice receiving preoperative TBT had increased water intake from BL to ICR (*p* < 0.05) when compared to CTR (Figure 1e). Relative to baseline weights, significant differences in percent weight change were observed between groups (Figure 1c). All groups demonstrated significant differences in percent weight change between ICR and W1 (*p* < 0.05), and between ICR and W2 (*p* < 0.05). No significant differences were observed at W3 for any groups receiving TBT supplementation or for all groups at W4, suggesting that TBT may facilitate a quicker weight regain following ICR.

### 3.2. ICR Imparts a Dramatic Shift in Microbial Ecology

**Figure 2:**
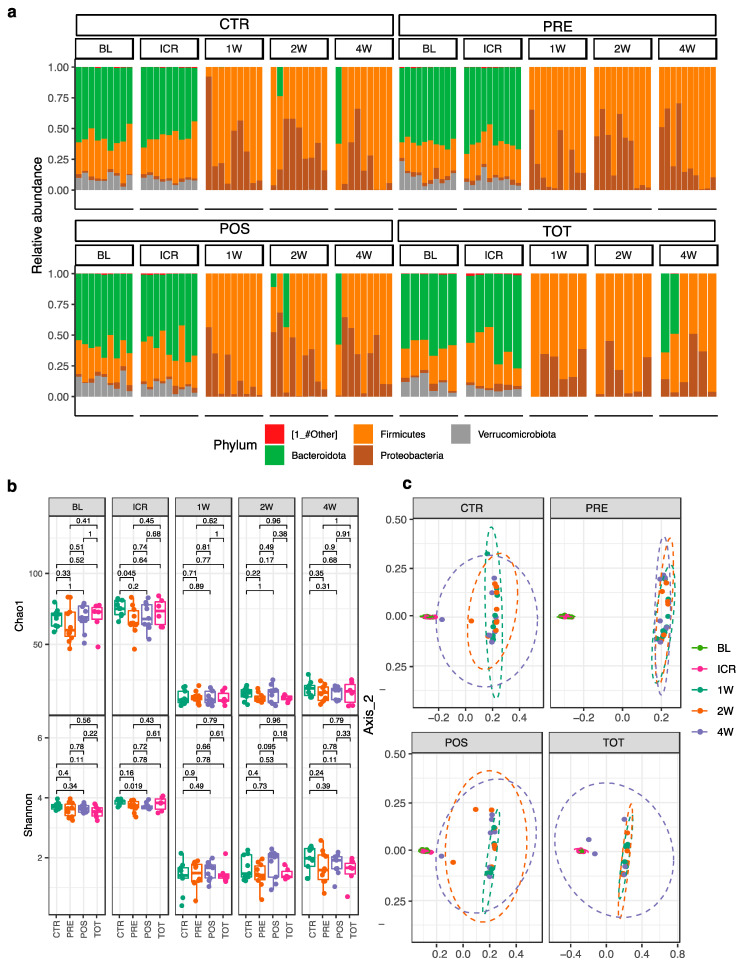
Difference in microbial abundance at the phylum level along changes in alpha and beta diversity from BL to W4. (**a**) Phylum-level differences in relative microbial abundance between groups over time. (**b**) Within- and between- group changes in α-diversity using Chao1 and Shannon indices. (**c**) Between-group differences from baseline to week 4 in β-diversity using weighted UniFrac analysis.

A dramatic shift in gut microbial ecology was observed in all groups following ICR (Supplemental Tables S1–S4). In decreasing order of relative abundance, the preoperative microbiome of all mice was composed of the following bacterial phyla: *Bacteroidetes*, *Firmicutes*, *Verrucomicrobia*, and *Proteobacteria* (Figure 2a). Immediately following ICR, at week 1, a complete loss of *Bacteroides* and *Verrucomicrobia* phyla was observed. These changes resulted in a corresponding bloom of *Firmicutes and Proteobacteria* that was sustained throughout the postoperative period. At four weeks, only four mice demonstrated a recovery of *Bacteroidetes*, three of which received postoperative TBT supplementation.

Evaluation of changes in α-diversity and in β-diversity revealed similar dramatic microbial changes imparted by ICR. α-diversity as assessed by Chao1 and Shannon indices significantly decreased from ICR to W1 and did not recover to preoperative levels at any time point. After one week of TBT supplementation (from BL to ICR), PRE mice demonstrated a decrease in alpha diversity (Figure 2b, *q* = 0.045) in comparison to CTR. No other differences in α-diversity were noted between groups at other time points. β-diversity analysis revealed a significant difference in pre- and postoperative microbial composition (Figure 2c, *q* < 0.05). Although, at four weeks, no intervention demonstrated a complete return to preoperative β-diversity, POS and TOT groups receiving postoperative TBT supplementation were associated with the most substantial shifts towards that of preoperative composition.

### 3.3. Timing of TBT Differential Modulates Recolonization Following ICR

**Figure 3:**
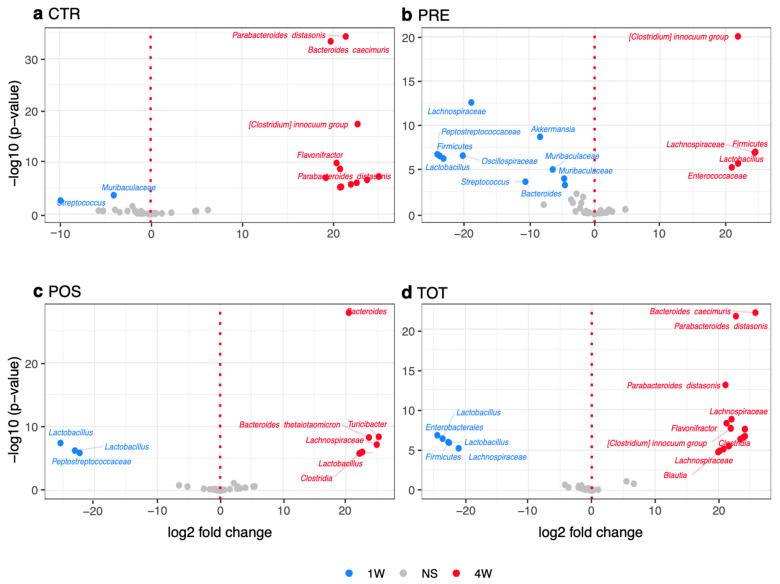
Volcano plots demonstrating significant differences (*p* adj. < 0.05) in relative abundance of microbial taxa from W1 to W4. (**a**) CTR group volcano plot. (**b**) PRE group. (**c**) POS group volcano plot. (**d**) TOT group volcano plot. NS represents values that are not significant.

Differences in the relative abundance of bacterial taxa between groups from W1 to W4 were assessed using DESeq2 to observe if TBT supplementation was able to facilitate a recolonization of anaerobic bacteria following ICR (Figure 3a–d). When comparing groups receiving preoperative supplementation to those receiving postoperative TBT, POS and TOT mice were associated with a significantly increased bloom of specific anaerobic taxa. These included *Bacteroides thetaiotaomicron*, *Bacteroides caecimuris*, *Parabacteroides distasonis*, *Clostridia*, and *Turicibacter.*

We next compared differences in relative abundance between groups at week 4 (Supplemental Figure S1). This analysis revealed that POS groups had a significant increase in *Clostridia* (Supplemental Figure S1, *p* < 0.05), a prominent anerobic class of commensal bacteria, while PRE groups had a relative bloom of *Staphylococcus*, *Lactobacillus*, and *Peptostreptococcacea.*

### 3.4. Effects of ICR and TBT on Fecal SCFA Concentrations

**Figure 4:**
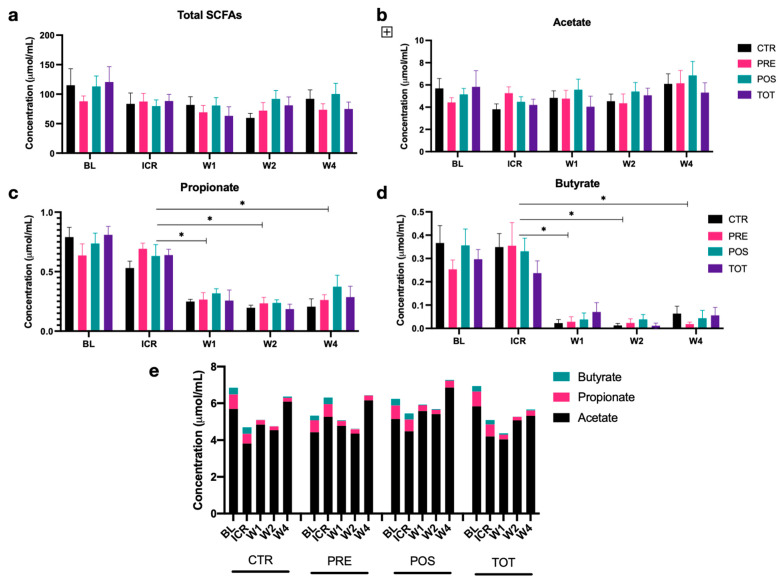
Concentrations of fecal short-chain fatty acids (SCFAs) from BL to W4 by intervention group. Column graphs represent median +/− SEM. (**a**) Total SCFAs. (**b**) Acetate. (**c**) Propionate. (**d**) Butyrate. (**e**) Proportion of SCFAs. * represents *p* < 0.05.

Neither total nor individual fecal SCFA concentrations differed significantly between groups from BL to W4 (Figure 4a–e). While acetate and total SCFA levels remained relatively unchanged during the course of the experiment, ICR resulted in a significant reduction in propionate and an even more pronounced loss of butyrate. Concentrations of both of these anti-inflammatory metabolites remained decreased from ICR to W4, with no return to preoperative levels observed in any group (Figure 4c–e). Notably, TBT supplementation was not found to alter fecal butyrate concentrations.

### 3.5. Perioperative Timing of TBT Differentially Modulates Gastrointestinal Inflammation

**Figure 5:**
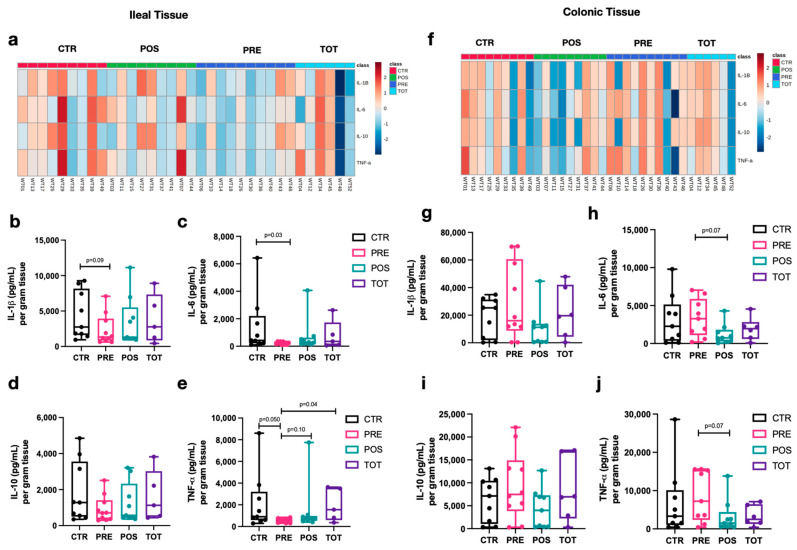
Tissue-weight-adjusted cytokine concentrations in perianastomotic ileal and colonic tissue homogenate. Box-and-whisker plots represent the distribution of each group at W4. The median is represented by the middle line while the upper and lower borders of the box plot identify the 75th and 25th percentile, respectively. The whiskers correspond to the maximal and minimal values. (**a**) Heatmap of ileal tissue cytokine concentrations after logarithmic transformation of data. (**b**–**e**) Concentration of IL-1 β, IL-6, IL-10, and TNF-α, respectively, per gram of dry ileal tissue. (**f**) Heatmap of colonic tissue cytokine concentrations after logarithmic transformation of data. (**g**–**j**) Concentration of IL-1 β, IL-6, IL-10, and TNF-α, respectively, per gram of dry colonic tissue.

Analysis of cytokine concentrations for perianastomotic ileal and colonic tissue at week 4 revealed different inflammatory profiles associated with TBT supplementation (Figure 5a–j). PRE mice had a trend towards decreased ileal inflammation as evidenced by decreased levels of IL-1β (Figure 5b, PRE vs. CTR; *p* = 0.09), IL-6 (Figure 5c, PRE vs. CTR *p* = 0.03), and TNF-α (Figure 5e PRE vs. CTR/TOT; *p* < 0.05). In contrast, POS mice demonstrated trends towards reduced colonic inflammation, particularly when comparing to PRE mice. This was demonstrated by decreased levels of IL-6 (Figure 5h, POS vs. PRE; *p* = 0.07) and TNF-α (Figure 5j POS vs. PRE; *p* = 0.07).

The correlation of cytokine trends observed in colonic tissue homogenate with the colonic weight to length ratio adds further support to the observation that TBT differentially modulates perianastomotic gastrointestinal inflammation. At week four, all groups demonstrated low levels of pro-inflammatory LPS (Supplemental Figure S2) and undetectable IL-6 in the serum, with no differences observed between groups.

### 3.6. TBT Supplementation Altered Colonic Tissue Weight to Length Ratio but Not Histologic Injury Scoring

Lastly, histologic injury scoring of ileal and colonic tissue revealed minimal levels of active tissue inflammation (Supplemental Figure S3a,b) and no significant differences between groups. Weight to length values of ileal tissue did not differ between groups (Supplemental Figure S3c). However, when comparing PRE mice with those receiving postoperative TBT (PRE vs. POS; *p* = 0.09; PRE vs. TOT *p* = 0.04), a trend towards reduced colonic weight to length ratio, in keeping with reduced tissue inflammation, was observed for postoperative TBT-supplemented mice (Supplemental Figure S3d), in keeping with our tissue homogenate cytokine findings discussed above.

## 4. Discussion

This study evaluated the effects of various perioperative tributyrin (TBT) regimens on gastrointestinal inflammation and gut microbial ecology using a mouse model of ileocecal resection. Preoperative TBT supplementation was associated with a reduction in ileal tissue inflammation and an increase in colonic tissue inflammation when compared to mice receiving postoperative TBT. The microbial recolonization of PRE mice was characterized by a bloom of *Staphylococcus*, *Lactobacillus*, *Enteroccaceae,* and *Peptostreptococcacea.* In contrast, mice treated with postoperative TBT had a bloom of anaerobes including *Bacteroides thetaiotaomicron*, *Bacteroides caecimuris*, *Parabacteroides distasonis*, *Clostridia*, and *Turicibacter*. Taken together, the results of our work demonstrate that the timing of TBT supplementation differentially modulates gastrointestinal inflammation and gut microbial recolonization following murine ICR.

Tributyrin is an oral butyrate analogue which has shown substantial promise in ameliorating inflammatory gastrointestinal pathology in murine studies. The first high-quality evidence was provided by Vinolo et al. nearly a decade ago using murine models of obesity and metabolic disease [26]. Supplementation of TBT in mice receiving a high-fat diet was found to attenuate systemic inflammation, insulin resistance, and hepatic steatosis. Subsequent studies by Cresci et al. on ethanol-induced murine gut injury further demonstrated a protective role for TBT in modulating gastrointestinal barrier integrity by increasing the expression of tight junction proteins in the ileum and proximal colon [21]. Lastly, elegant work by Rivera-Chavez et al. showed that TBT was able to restore the epithelial anaerobic environment perturbed by streptomycin treatment and prevent an aerobic expansion of pathogenic Salmonella enterica [27].

In healthy colonic mucosa, butyrate inhibits histone deacetylases, leading to reduced activation of the pro-inflammatory NF-kB pathway, increased expression of tight junction proteins which promote gut barrier integrity, regulation of antimicrobial peptides, and attenuation of aberrant innate and adaptive host immune responses. Additional in vivo evidence in support of butyrate and SCFA supplementation as a promising target for restoring microbial-mediated intestinal epithelial dysfunction exists. For example, administration of oral short-chain fatty acids has been found to restore the abnormal intestinal epithelial cell turnover present in specific pathogen-free mice after antibiotic depletion of SCFA producing bacterial taxa. Given the well-accepted anti-inflammatory properties of butyrate on the intestinal epithelium, our findings of decreased ileal inflammation at the expense of increased colonic inflammation when comparing mice receiving preoperative and postoperative supplementation are unexpected.

While our study was not specifically designed to evaluate the underlying mechanisms responsible for these findings, a number of possible explanations exist. The degradation of TBT, release of butyrate, and subsequent absorption of butyrate along the length of the gastrointestinal tract are currently not well understood. It is thus possible that in the PRE group, TBT may have been preferentially absorbed in the proximal small intestine prior to reaching the colon and providing any potential benefits. Removal of the terminal ileum, resulting in bile acid changes and altered gastrointestinal motility [28], may have facilitated an increased delivery of butyrate to mice supplemented TBT postoperatively, leading to improved colonic inflammatory profiles.

Alternatively, it is possible that the timing of TBT in relation to ICR may have altered the concentrations of butyrate at the level of the intestinal crypts to modulate tissue inflammatory responses. Butyrate exerts differential effects on intestinal crypt stem cells by impairing cell proliferation in response to mucosal injury through Foxp3 transcription factor-dependent mechanisms [29]. Supra-physiologic butyrate concentrations in the presence of healthy colonic epithelial cells may have overcome endogenous colonocyte butyrate utilization capacity. This would in turn increase crypt concentrations in the PRE and TOT groups, leading to impaired responses to surgical insult. In POS mice which received only postoperative supplementation, however, these supra-physiologic butyrate concentrations may have instead been fully utilized by injured colonocytes for restoration of barrier integrity and immune regulation.

The advent of high-throughput, cost-effective sequencing technologies has brought a remarkable understanding of the complex gut microbial ecologic shifts imparted by surgery. However, our ability to modulate these shifts, including restoring the loss of anti-inflammatory SCFA-producing anaerobes, in the perioperative period has been met with initial challenges. Through stimulation of hypoxia inducible factor-1 (HIF-1), butyrate presents a particularly promising strategy as it has been demonstrated to restore luminal hypoxia via regulation of enterocyte transcriptional factors and improve gut barrier integrity in colitis models [22,30]. Findings from our study indeed support the promise of this concept. All mice receiving postoperative TBT demonstrated an increased bloom of anaerobic bacteria, with the TOT group showing the greatest bloom and also a trend towards the restoration of beta diversity at four weeks.

Our study was not without its limitations. Mice used in our experiment did not have active gastrointestinal inflammation at the time of surgery, which may have limited our ability to observe significant differences in inflammatory markers between intervention groups. In future studies, this limitation could be overcome either by using mouse colitis models, or by separating arms into early and late cohorts. Early cohorts, between 1 and 3 weeks postoperatively, may allow for a clearer evaluation of inflammatory markers and for changes in gross histology. Late cohorts, on the other hand, may provide more complete characterization regarding the timing and effect of TBT on postoperative microbial recolonization. Additional limitations were that TBT dosing was provided through preexisting water supplies, making dosing less accurate than oral gavage. However, the stress of surgery combined with that of daily gavage makes the use of the gavage vehicle difficult to justify. Lastly, our study did not elucidate the exact mechanisms responsible for our immunologic or microbial findings and should thus serve as hypothesis generating.

Despite these limitations, this study is the first to demonstrate that the perioperative timing of a supplement aimed at optimizing the gut microbiome differentially alters gastrointestinal inflammation and gut microbial recolonization following ileocecal resection. Results of this study build upon our understanding of perioperative gut microbial shifts and provide evidence for the ongoing pursuit of gut microbial modulation strategies as a novel therapeutic modality following gastrointestinal surgery. Further, the fact that our murine ICR mirrors that of ICR performed in human patients suffering from CD provides a practical framework from which to translate the findings from this basic biological mouse study to real-world clinical applications.

## 5. Conclusions

The timing of tributyrin supplementation differentially modulates gastrointestinal inflammation and gut microbial recolonization following murine ileocecal resection. These findings build upon our understanding of perioperative gut microbial shifts and provide evidence for the ongoing pursuit of gut microbial modulation strategies as a novel therapeutic modality following gastrointestinal surgery.

## Data Availability

The data presented in this study are available on request from the corresponding author.

## Supplementary Materials

The following are available online at https://www.mdpi.com/article/10.3390/nu13062069/s1, Figure S1: Volcano plots demonstrating differences in relative abundance between groups at W4 using DESeq2 analysis. Figure S2: Differences in serum LPS between groups at W4. Box-and-whisker plots represent the distribution of each group at W4. Figure S3: Perianastomotic ileal and colonic tissue histologic injury scoring and weight to length ratios. Table S1: Shifts in microbial taxa from BL to W4 in CTR mice. Table S2: Shifts in microbial taxa from BL to W4 in PRE mice. Table S3: Shifts in microbial taxa from BL to W4 in POS mice. Table S4: Shifts in microbial taxa from BL to W4 in TOT mice.
