# Supplementary material for: Timing of Tributyrin Supplementation Differentially Modulates Gastrointestinal Inflammation and Gut Microbial Recolonization Following Murine Ileocecal Resection
Source: Nutrients. 2021 Jun 17;13(6):2069. doi: 10.3390/nu13062069 (PMC8233937; doi:10.3390/nu13062069)
Supplement: Supplementary file 1 [file nutrients-13-02069-s001.zip › nutrients-1263887-supplementary.pdf]

CTR Group		Time Point				
		BL	ICR	W1	W2	W4
**Phylum**						
	Bacteroidetes	58.0 (2.2)	56.0 (2.0)	0.03 (0.03)	2.7 (2.5)	6.9 (6.9)
	Firmicutes	29.9 (2.7)	33.5 (2.6)	68.1 (9.7)	64.8 (6.3)	75.3 (8.7)
	Proteobacteria	2.1 (0.3)	2.2 (0.2)	31.9 (9.7)	32.5 (6.6)	17.7 (7.6)
	Verrucomicrobiota	9.7 (1.3)	8.0 (0.8)	0 (0)	0.02 (0.02)	0 (0)
**Class**						
	Alphaproteobacteria	1.0 (0.2)	1.1 (0.2)	0 (0)	0.01 (0.01)	0 (0)
	Bacilli	3.5 (0.7)	3.2 (0.8)	47.7 (8.9)	48.3 (6.1)	44.8 (7.1)
	Bacteroidia	58.0 (2.2)	56.0 (2.0)	0.03 (0.03)	2.7 (2.6)	7.1 (7.1)
	Clostridia	26.3 (2.7)	30.2 (2.2)	20.1 (7.1)	16.1 (7.7)	29.7 (7.0)
	Gammaproteobacteria	1.1 (0.1)	1.1 (0.1)	32.2 (9.7)	33.0 (6.6)	18.4 (7.8)
	Verrucomicrobiae	9.7 (1.3)	8.0 (0.8)	0 (0)	0.02 (0.02)	0 (0)
**Family**						
	Akkermansiaceae	9.9 (1.3)	8.1 (0.8)	0 (0)	0.02 (0.02)	0 (0)
	Bacteroidaceae	7.5 (0.7)	7.1 (0.8)	0 (0)	0 (0)	6.3 (6.3)
	Clostridiaceae	0 (0)	0 (0)	3.6 (3.6)	8.3 (8.3)	8.4 (8.4)
	Enterobacteriaceae	0.01 (0.01)	0 (0)	30.6 (9.5)	33.0 (6.7)	18.1 (7.6)
	Enterococcaceae	0 (0)	0 (0)	4.1 (1.4)	8.0 (1.8)	9.6 (2.1)
	Erysipelotrichaceae	0.2 (0.1)	0.1 (0.04)	0.8 (0.6)	2.5 (1.8)	5.2 (3.3)
	Lachnospiraceae	21.8 (2.2)	25.6 (2.3)	0.01 (0.01)	0.3 (0.3)	3.6 (2.3)
	Lactobacillaceae	2.5 (0.7)	2.3 (0.9)	44.0 (8.9)	37.7 (5.0)	30.7 (5.9)
	Muribaculaceae	47.4 (2.1)	46.4 (1.8)	0.03 (0.03)	0 (0)	0 (0)
	Oscillospiraceae	2.9 (0.6)	3.2 (0.3)	0 (0)	0.3 (0.3)	0.2 (0.2)
	Peptostreptococcaceae	0.01 (0.01)	0.01 (0.01)	16.5 (7.3)	7.1 (2.7)	17.1 (3.7)
	Sutterellaceae	1.1 (0.1)	1.1 (0.1)	0 (0)	0 (0)	0 (0)
	Tannerellaceae	3.9 (0.6)	3.1 (0.4)	0 (0)	2.7 (2.7)	0.9 (0.9)
**Genus**						
	[Clostridium] inoculum group	0.4 (0.2)	0.3 (0.1)	0 (0)	0.6 (0.4)	1.7 (0.7)
	Akkermansia	32.3 (4.0)	28.2 (2.4)	0 (0)	0.02 (0.02)	0 (0)
	Anaeroplasma	2.7 (1.0)	2.6 (1.1)	0 (0)	0 (0)	0 (0)
	ASF356	2.0 (0.4)	3.0 (0.9)	0 (0)	0 (0)	0 (0)
	Bacteroides	0 (0)	24.9 (2.6)	0 (0)	0 (0)	7.3 (7.3)
	Clostridium sensu stricto 1	0 (0)	0 (0)	2.9 (2.9)	8.2 (8.2)	8.3 (8.3)
	Enterococcus	0 (0)	0 (0)	5.1 (1.8)	9.7 (2.0)	13.1 (2.9)
	Escherichia-Shigella	0.02 (0.02)	0 (0)	17.9 (6.3)	16.6 (5.9)	8.6 (4.7)

Lachnospiraceae NK4A136 group	2.4 (1.0)	4.2 (1.5)	0 (0)	0 (0)	0 (0)
Lactobacillus	7.9 (2.4)	7.8 (2.7)	72.3 (7.8)	59.3 (8.5)	53.7 (9.7)
Muribaculum	2.8 (1.0)	4.1 (1.3)	0 (0)	0 (0)	0 (0)
Parabacteroides	12.3 (1.9)	10.6 (1.4)	0 (0)	2.9 (2.9)	1.0 (1.0)
Parasutterella	3.5 (0.4)	3.9 (0.2)	0 (0)	0 (0)	0 (0)
Turicibacter	0 (0)	0 (0)	1.0 (0.7)	2.2 (1.7)	6.2 (3.9)

Supplemental Table 1. Shifts in microbial taxa from BL to W4 in CTR mice. Values reported as median and standard error measurement.

PRE Group		Time Point				
		BL	ICR	W1	W2	W4
Phylum						
	Bacteroidetes	61.2 (0.9)	60.8 (2.2)	0 (0)	0 (0)	0 (0)
	Firmicutes	24.6 (2.0)	28.1 (2.0)	79.0 (6.9)	68.3 (7.8)	73.7 (8.3)
	Proteobacteria	3.4 (0.7)	3.0 (0.3)	20.9 (6.9)	31.7 (7.8)	26.3 (8.3)
	Verrucomicrobiota	10.4 (1.9)	7.8 (1.4)	0 (0)	0 (0)	0 (0)
Class						
	Alphaproteobacteria	1.9 (0.6)	1.5 (0.3)	0 (0)	0 (0)	0 (0)
	Bacilli	3.3 (1.0)	2.4 (0.4)	55.8 (6.2)	58.3 (6.9)	56.9 (7.6)
	Bacteroidia	61.2 (0.9)	60.8 (2.2)	0 (0)	0 (0)	0 (0)
	Clostridia	21.3 (2.0)	25.7 (2.1)	23.0 (8.4)	9.5 (3.1)	16.3 (5.4)
	Gammaproteobacteria	1.5 (0.1)	1.5 (0.2)	21.1 (6.9)	32.3 (7.9)	26.8 (8.3)
	Verrucomicrobiae	10.4 (1.9)	7.8 (1.4)	0 (0)	0 (0)	0 (0)
Family						
	Akkermansiaceae	10.8 (1.9)	8.0 (1.4)	0 (0)	0 (0)	0 (0)
	Bacteroidaceae	12.6 (1.8)	11.5 (1.3)	0 (0)	0 (0)	0 (0)
	Clostridiaceae	0 (0)	0 (0)	0 (0)	0.1 (0.1)	0 (0)
	Enterobacteriaceae	0.1 (0.1)	0.02 (0.02)	21.1 96.9)	31.4 (7.7)	26.4 (8.4)
	Enterococcaceae	0 (0)	0 (0)	1.9 (0.9)	4.6 (1.3)	7.2 (1.3)
	Erysipelotrichaceae	0.2 (0.1)	0.08 (0.03)	4.1 (3.1)	5.7 (3.6)	10.4 (5.8)
	Lachnospiraceae	17.9 (2.1)	21.1 (2.2)	0 (0)	1.0 (1.0)	3.0 (2.0)
	Lactobacillaceae	2.6 (1.0)	1.8 (0.4)	49.6 (5.8)	48.8 (8.0)	40.0 (8.6)
	Muribaculaceae	45.2 (1.5)	45.4 (2.1)	0 (0)	0 (0)	0 (0)
	Oscillospiraceae	2.0 (0.4)	2.9 (0.6)	0 (0)	0 (0)	0.2 (0.2)
	Peptostreptococcaceae	0.04 (0.03)	0.1 (0.03)	22.9 (8.4)	8.4 (2.8)	12.8 (4.4)
	Sutterellaceae	1.5 (0.1)	1.4 (0.2)	0 (0)	0 (0)	0 (0)
	Tannerellaceae	5.8 (0.9)	5.4 (0.2)	0 (0)	0 (0)	0 (0)
Genus						
	[Clostridium] inoculum group	0.4 (0.1)	0.2 (0.1)	0 (0)	0.8 (0.5)	1.7 (0.9)
	Akkermansia	28.2 (4.4)	24.3 (3.6)	0 (0)	0 (0)	0 (0)
	Anaeroplasma	1.8 (0.8)	2.0 (1.1)	0 (0)	0 (0)	0 (0)
	ASF356	1.4 (0.3)	3.5 (0.9)	0 (0)	0 (0)	0 (0)
	Bacteroides	33.4 (3.2)	33.8 (2.4)	0 (0)	0 (0)	0 (0)
	Clostridium sensu stricto 1	0 (0)	0 (0)	0.1 (0.1)	0.1 (0.1)	0 (0)
	Enterococcus	0 (0)	0 (0)	2.7 (1.1)	5.3 (1.1)	10.0 (2.9)
	Escherichia-Shigella	0.1 (0.1)	0 (0)	16.6 (5.2)	12.2 (5.6)	8.6 (3.6)
	Lachnospiraceae NK4A136 group	1.3 (0.7)	1.3 (0.7)	0 (0)	0 (0)	0 (0)
	Lactobacillus	6.9 (2.3)	5.1 (0.8)	75.8 (6.3)	75.4 (8.7)	65.2 (8.8)

Muribaculum	1.8 (1.0)	2.2 (1.1)	0 (0)	0 (0)	0 (0)
Parabacteroides	15.2 (1.8)	15.9 (1.8)	0 (0)	0 (0)	0 (0)
Parasutterella	4.1 (0.3)	4.4 (0.5)	0 (0)	0 (0)	0 (0)
Turicibacter	0 (0)	0 (0)	4.3 (3.1)	6.1 (3.7)	14.0 (7.1)

Supplemental Table 2. Shifts in microbial taxa from BL to W4 in PRE mice. Values reported as median and standard error measurement.

POS Group		Time Point				
		BL	ICR	W1	W2	W4
Phylum						
	Bacteroidetes	59.6 (2.2)	58.5 (3.7)	0 (0)	6.1 (4.9)	6.4 (6.4)
	Firmicutes	24.7 (2.5)	30.2 (3.9)	82.9 (6.7)	64.7 (8.2)	64.2 (7.8)
	Proteobacteria	3.0 (0.5)	2.7 (0.6)	17.1 (6.7)	29.2 (8.2)	29.4 (8.1)
	Verrucomicrobiota	12.5 (1.8)	8.1 (1.4)	0 (0)	0 (0)	0 (0)
Class						
	Alphaproteobacteria	1.6 (0.4)	1.6 (0.5)	0 (0)	0 (0)	0 (0)
	Bacilli	2.6 (0.5)	1.7 (0.4)	62.7 (6.4)	47.4 (6.0)	46.7 (7.4)
	Bacteroidia	59.4 (2.2)	58.5 (3.7)	0 (0)	6.3 (5.1)	6.5 (6.5)
	Clostridia	22.1 (2.6)	28.4 (4.1)	20.1 (8.5)	16.9 (6.2)	16.9 (6.5)
	Gammaproteobacteria	1.4 (0.3)	1.1 (0.1)	17.2 (6.6)	29.4 (8.2)	29.9 (8.1)
	Verrucomicrobiae	12.5 (1.8)	8.1 (1.4)	0 (0)	0 (0)	0 (0)
Family						
	Akkermansiaceae	12.9 (1.9)	8.3 (1.4)	0 (0)	0 (0)	0 (0)
	Bacteroidaceae	9.7 (1.9)	9.4 (1.6)	0 (0)	6.4 (5.1)	6.7 (6.7)
	Clostridiaceae	0 (0)	0 (0)	0 (0)	0.7 (0.7)	0 (0)
	Enterobacteriaceae	0.1 (0)	0 (0)	14.0 (6.4)	16.5 (6.7)	29.7 (8.1)
	Enterococcaceae	0 (0)	0 (0)	3.9 (1.5)	8.2 (0.9)	8.9 (3.5)
	Erysipelotrichaceae	0.2 (0.1)	0.1 (0)	2.8 (2.7)	3.2 (1.9)	3.8 (1.9)
	Lachnospiraceae	18.5 (2.7)	24.3 (4.0)	0 (0)	0.5 (0.5)	2.3 (1.3)
	Lactobacillaceae	2.1 (0.6)	1.4 (0.4)	58.5 (7.6)	49.4 (8.5)	34.4 (4.0)
	Muribaculaceae	46.8 (1.7)	46.5 (3.1)	0 (0)	0 (0)	0 (0)
	Oscillospiraceae	2.0 (0.4)	2.7 (0.6)	0 (0)	0.1 (0)	0 (0)
	Peptostreptococcaceae	0.1 (0)	0.1 (0)	20.0 (8.5)	15.1 (5.6)	14.1 (6.4)
	Sutterellaceae	1.3 (0.2)	1.2 (0.1)	0 (0)	0 (0)	0 (0)
	Tannerellaceae	5.2 (0.6)	4.4 (0.5)	0 (0)	0 (0)	0 (0)
Genus						
	[Clostridium] inoculum group	0.3 (0.1)	0.2 (0.1)	0.2 (0.2)	1.8 (1.3)	1.6 (0.6)
	Akkermansia	37.7 (5.0)	29.4 (4.7)	0 (0)	0 (0)	0 (0)
	Anaeroplasma	1.4 (0.7)	0.9 (0.6)	0 (0)	0 (0)	0 (0)
	ASF356	2.3 (0.7)	4.4 (1.3)	0 (0)	0 (0)	0 (0)
	Bacteroides	27.3 (4.3)	31.5 (4.1)	0 (0)	11.4 (7.6)	8.5 (8.5)
	Clostridium sensu stricto 1	0 (0)	0 (0)	0 (0)	1.1 (1.0)	0 (0)
	Enterococcus	0 (0)	0.1 (0)	4.9 (2.1)	9.0 (1.1)	11.1 (3.3)
	Escherichia-Shigella	0 (0)	0 (0)	14.4 (6.3)	11.6 (5.1)	14.6 (6.3)
	Lachnospiraceae NK4A136 group	1.2 (0.6)	0.7 (0.5)	0 (0)	0 (0)	0 (0)
	Lactobacillus	5.6 (1.3)	4.8 (1.4)	77.0 (7.0)	62.4 (8.2)	60.8 (8.7)

Muribaculum	0.7 (0.7)	0.7 (0.7)	0 (0)	0 (0)	0 (0)
Parabacteroides	14.7 (1.3)	15.3 (1.5)	0 (0)	0 (0)	0 (0)
Parasutterella	3.5 (0.6)	4.1 (0.2)	0 (0)	0 (0)	0 (0)
Turicibacter	0 (0)	0 (0)	2.7 (2.7)	2.4 (1.9)	3.3 (1.6)

Supplemental Table 3. Shifts in microbial taxa from BL to W4 in POS mice. Values reported as median and standard error measurement.

TOT Group		Time Point				
		BL	ICR	W1	W2	W4
**Phylum**						
	Bacteroidetes	58.8 (2.0)	59.4 (5.5)	0 (0)	0 (0)	18.8 (12.1)
	Firmicutes	27.1 (2.2)	30.4 (5.9)	77.4 (6.1)	82.1 (7.4)	61.0 (11.3)
	Proteobacteria	2.7 (0.7)	3.1 (0.7)	22.6 (6.1)	17.9 (7.4)	20.2 (7.9)
	Verrucomicrobiota	11.0 (2.5)	6.1 (0.7)	0 (0)	0 (0)	0 (0)
**Class**						
	Alphaproteobacteria	1.4 (0.6)	2.0 (0.6)	0 (0)	0 (0)	0 (0)
	Bacilli	3.6 (1.3)	2.8 (0.8)	54.4 (6.9)	65.1 (5.9)	48.0 (13.4)
	Bacteroidia	58.9 (2.0)	59.5 (5.5)	0 (0)	0 (0)	18.9 (12.1)
	Clostridia	23.4 (3.0)	27.6 (5.6)	22.6 (10.6)	16.9 (8.4)	12.6 (4.1)
	Gammaproteobacteria	1.3 (0.2)	1.2 (0.1)	23.0 (6.2)	18.0 (7.4)	20.4 (8.1)
	Verrucomicrobiae	11.0 (2.6)	6.1 (0.7)	0 (0)	0 (0)	0 (0)
**Family**						
	Akkermansiaceae	11.4 (2.6)	6.4 (0.8)	0 (0)	0 (0)	0 (0)
	Bacteroidaceae	15.0 (3.1)	13.1 (4.1)	0 (0)	0 (0)	9.3 (9.3)
	Clostridiaceae	0 (0)	0 (0)	0 (0)	0 (0)	0 (0)
	Enterobacteriaceae	0 (0)	0 (0)	22.4 (5.9)	18.0 (7.5)	20.1 (8.0)
	Enterococcaceae	0 (0)	0 (0)	0 (0.0)	9.4 (6.2)	3.1 (1.0)
	Erysipelotrichaceae	0.1 (0.1)	0 (0)	0 (0)	0.2 (0.2)	0.4 (0.3)
	Lachnospiraceae	19.5 (3.5)	22.9 (4.7)	0 (0)	0 (0)	5.1 (2.7)
	Lactobacillaceae	2.5 (1.0)	1.3 (0.4)	53.4 (6.9)	56.0 (10.0)	44.6 (13.1)
	Muribaculaceae	40.0 (3.3)	42.4 (2.1)	0 (0)	0 (0)	0 (0)
	Oscillospiraceae	1.7 (0.5)	2.6 (1.0)	0 (0)	0 (0)	0.2 (0.2)
	Peptostreptococcaceae	0.1 (0.1)	0 (0)	22.6 (10.6)	16.5 (8.1)	7.1 (2.8)
	Sutterellaceae	1.4 (0.2)	1.2 (0.1)	0 (0)	0 (0)	0 (0)
	Tannerellaceae	5.4 (1.4)	5.4 (1.5)	0 (0)	0 (0)	10.0 (8.2)
**Genus**						
	[Clostridium] inoculum group	0.2 (0.1)	0.3 (0.1)	0 (0)	0.3 (0.3)	0.5 (0.3)
	Akkermansia	28.0 (7.0)	19.0 (3.1)	0 (0)	0 (0)	0 (0)
	Anaeroplasma	2.4 (0.8)	3.9 (1.2)	0 (0)	0 (0)	0 (0)
	ASF356	2.4 (0.9)	2.6 (1.1)	0 (0)	0 (0)	0 (0)
	Bacteroides	35.8 (7.1)	34.2 (7.7)	0 (0)	0 (0)	12.8 (12.8)
	Clostridium sensu stricto 1	0 (0)	0 (0)	0 (0)	0 (0)	0 (0)
	Enterococcus	0 (0)	0 (0)	1.6 (1.2)	14.5 (11.0)	3.1 (0.8)
	Escherichia-Shigella	0 (0)	0 (0)	16.7 (8.4)	11.2 (5.7)	8.4 (5.8)
	Lachnospiraceae NK4A136 group	1.9 (1.1)	3.8 (1.8)	0 (0)	0 (0)	0 (0)
	Lactobacillus	5.7 (2.2)	4.0 (1.4)	81.1 (9.0)	74.0 (10.9)	61.2 (16.0)

Muribaculum	1.6 (1.1)	2.8 (1.8)	0 (0)	0 (0)	0 (0)
Parabacteroides	12.5 (3.3)	14.1 (2.7)	0 (0)	0 (0)	11.8 (9.3)
Parasutterella	3.2 (0.3)	3.6 (0.3)	0 (0)	0 (0)	0 (0)
Turicibacter	0 (0)	0 (0)	0 (0)	0 (0)	0 (0)

Supplemental Table 4. Shifts in microbial taxa from BL to W4 in TOT mice. Values reported as median and standard error measurement.

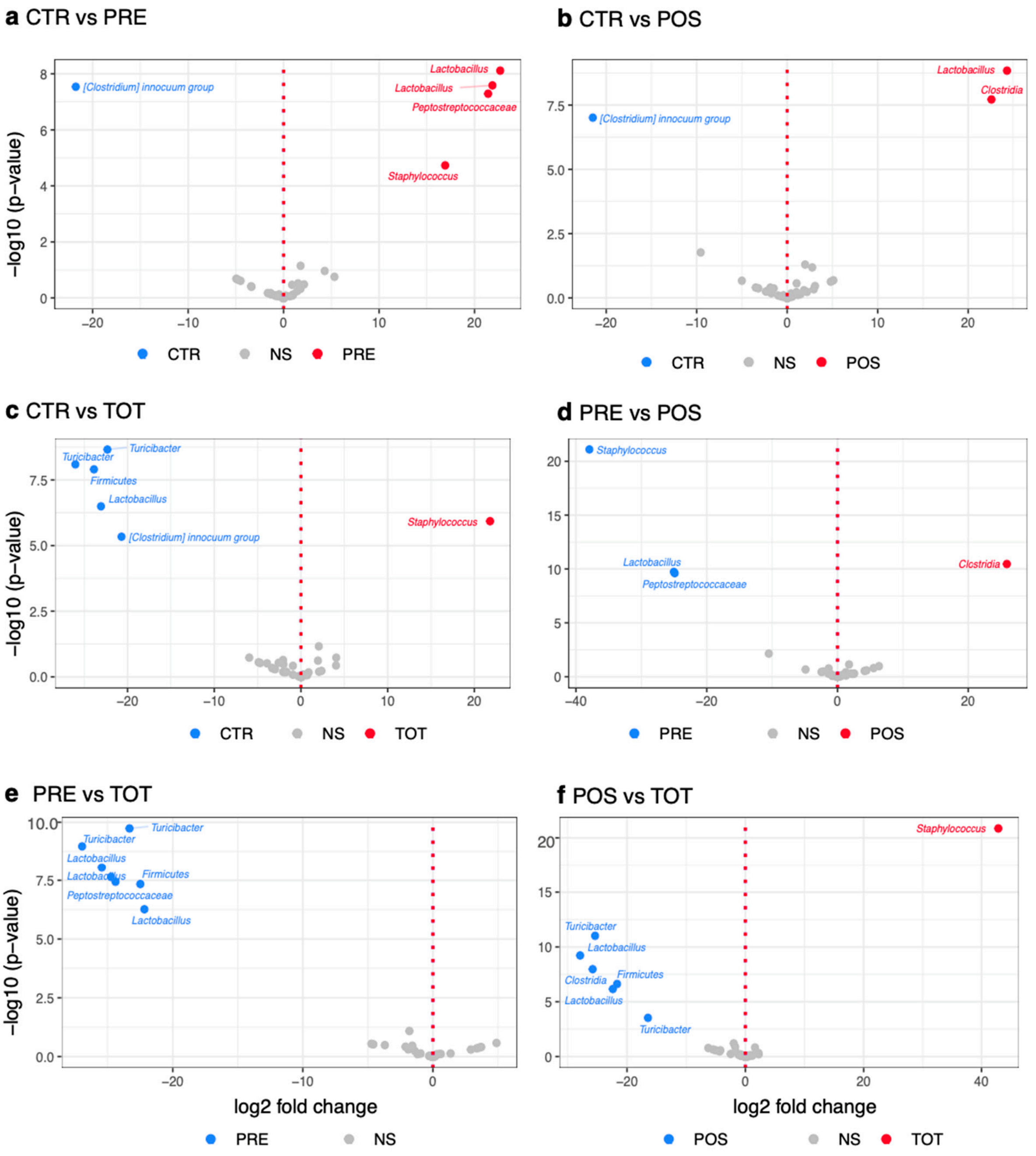

Supplemental Figure 1. Volcano plots demonstrating differences in relative abundance between groups at W4 using DESeq2 analysis. a. CTR vs. PRE. **b.** CTR vs. POS. **c.** CTR vs. TOT. **d.** PRE vs. TOT **e.** PRE vs TOT **f.** POS vs. TOT.

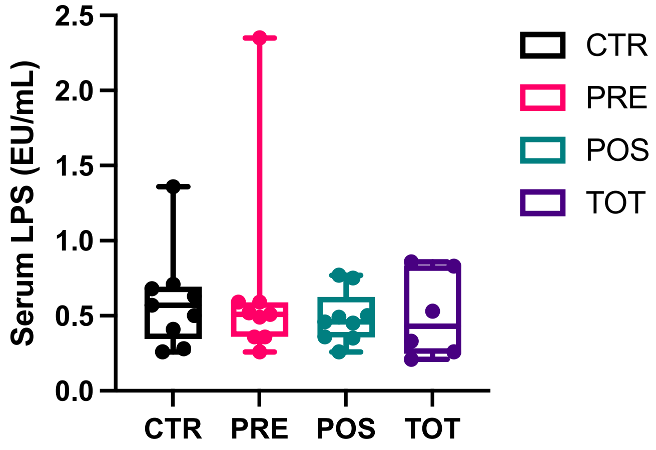

Supplemental Figure 2. Differences in serum LPS between groups at W4. Box-and-whisker plots represent the distribution of each group at W4. The median is represented by the middle line while the upper and lower borders of the box plot identify the 75th and 25th percentile, respectively. The whiskers correspond to the maximal and minimal values.

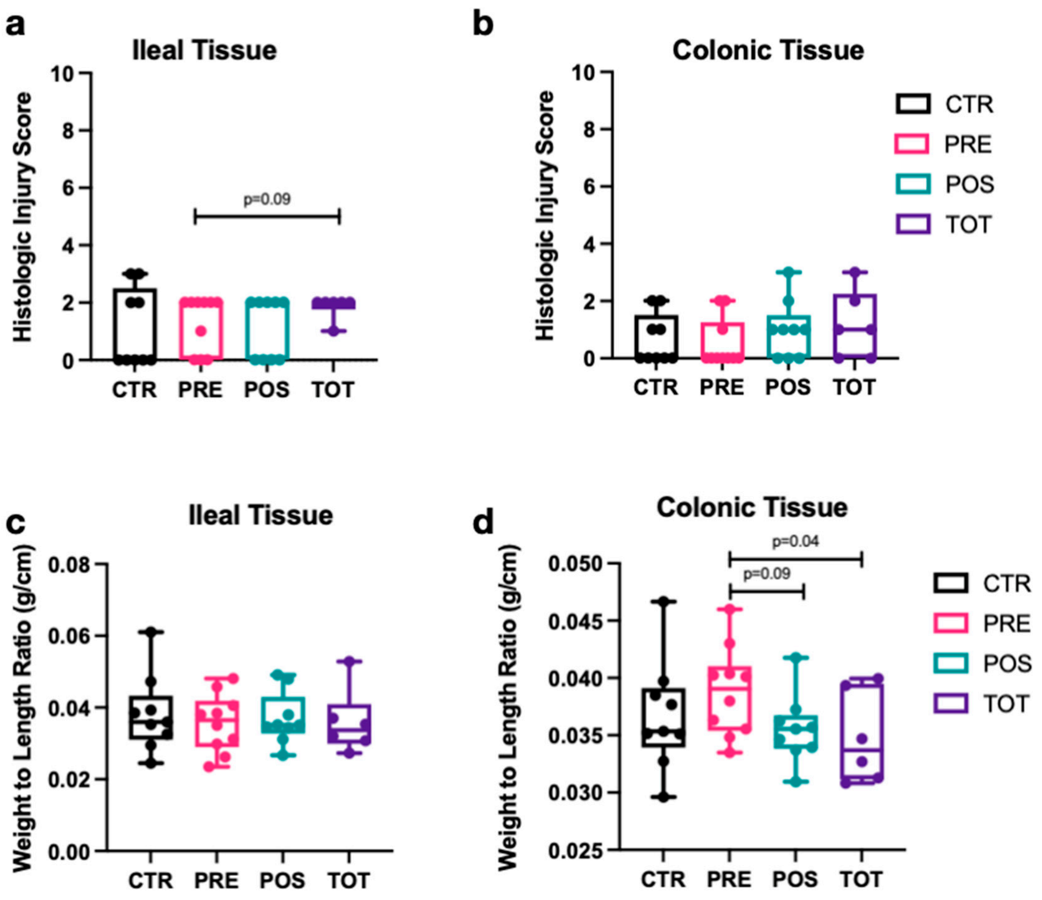

Supplemental Figure 3. Perianastomotic ileal and colonic tissue histologic injury scoring and weight to length ratios. Box-and-whisker plots represent the distribution of each group at week 4. The median is represented by the middle line while the upper and lower borders of the box plot identify the 75th and 25th percentile, respectively. The whiskers correspond to the maximal and minimal values. **a-b.** Histologic injury scoring for ileal and colonic tissues, respectively. **c-d.** Weight to length ratio for ileal and colonic tissue, respectively.
